# Whole-genome sequencing and identification of *Morganella morganii *KT pathogenicity-related genes

**DOI:** 10.1186/1471-2164-13-S7-S4

**Published:** 2012-12-07

**Authors:** Yu-Tin Chen, Hwei-Ling Peng, Wei-Chung Shia, Fang-Rong Hsu, Chuian-Fu Ken, Yu-Ming Tsao, Chang-Hua Chen, Chun-Eng Liu, Ming-Feng Hsieh, Huang-Chi Chen, Chuan-Yi Tang, Tien-Hsiung Ku

**Affiliations:** 1 Department of Computer Science, National Tsing Hua University, 101, Section 2, Kuang-Fu Rd., Hsinchu, Taiwan; 2 Department of Biological Science and Technology, National Chiao Tung University, 1001, University Road, Hsinchu, Taiwan; 3 Cancer Research Center, Changhua Christian Hospital, 135, Nanhsiao St., Changhua, Taiwan; 4 Master's Program in Biomedical Informatics and Biomedical Engineering, Feng Chia University, 100 Wenhwa Rd., Taichung, Taiwan; 5 Department of Information Engineering and Computer Sciences, Feng Chia University, 100 Wenhwa Rd., Taichung, Taiwan; 6 Institute of Biotechnology, National Changhua University of Education, 2 Shi-Da Rd., Changhua, Taiwan; 7 Department of Anesthesiology, Changhua Christian Hospital, 135, Nanhsiao St., Changhua, Taiwan; 8 The Division of Infectious Diseases, Department of Internal Medicine, Changhua Christian Hospital, 135, Nanhsiao St., Changhua, Taiwan; 9 Division of Critical Care Medicine, Department of Internal Medicine, Changhua Christian Hospital, 135, Nanhsiao St., Changhua, Taiwan; 10 Department of Computer Science, Providence University, 200, Chung-Chi Rd., Taichung, Taiwan

## Abstract

**Background:**

The opportunistic enterobacterium, *Morganella morganii*, which can cause bacteraemia, is the ninth most prevalent cause of clinical infections in patients at Changhua Christian Hospital, Taiwan. The KT strain of *M. morganii *was isolated during postoperative care of a cancer patient with a gallbladder stone who developed sepsis caused by bacteraemia. *M. morganii *is sometimes encountered in nosocomial settings and has been causally linked to catheter-associated bacteriuria, complex infections of the urinary and/or hepatobiliary tracts, wound infection, and septicaemia. *M. morganii *infection is associated with a high mortality rate, although most patients respond well to appropriate antibiotic therapy. To obtain insights into the genome biology of *M. morganii *and the mechanisms underlying its pathogenicity, we used Illumina technology to sequence the genome of the KT strain and compared its sequence with the genome sequences of related bacteria.

**Results:**

The 3,826,919-bp sequence contained in 58 contigs has a GC content of 51.15% and includes 3,565 protein-coding sequences, 72 tRNA genes, and 10 rRNA genes. The pathogenicity-related genes encode determinants of drug resistance, fimbrial adhesins, an IgA protease, haemolysins, ureases, and insecticidal and apoptotic toxins as well as proteins found in flagellae, the iron acquisition system, a type-3 secretion system (T3SS), and several two-component systems. Comparison with 14 genome sequences from other members of *Enterobacteriaceae *revealed different degrees of similarity to several systems found in *M. morganii*. The most striking similarities were found in the IS4 family of transposases, insecticidal toxins, T3SS components, and proteins required for ethanolamine use (*eut *operon) and cobalamin (vitamin B_12_) biosynthesis. The *eut *operon and the gene cluster for cobalamin biosynthesis are not present in the other *Proteeae *genomes analysed. Moreover, organisation of the 19 genes of the *eut *operon differs from that found in the other non-*Proteeae *enterobacterial genomes.

**Conclusions:**

This is the first genome sequence of *M. morganii*, which is a clinically relevant pathogen. Comparative genome analysis revealed several pathogenicity-related genes and novel genes not found in the genomes of other members of *Proteeae*. Thus, the genome sequence of *M. morganii *provides important information concerning virulence and determinants of fitness in this pathogen.

## Background

The Gram-negative anaerobic rod *Morganella morganii *is the only species in the genus *Morganella*, which belongs to the tribe *Proteeae *of the family *Enterobacteriaceae*. The other genera in the tribe *Proteeae *are *Proteus *and *Providencia*. Species belonging to *Morganella, Proteus*, and *Providencia *are found in the environment and as part of the normal flora of humans. They are also important opportunistic pathogens, which cause a wide variety of nosocomial infections following surgery. Reports of individual cases of infection and nosocomial-outbreaks have revealed that infection with *M. morganii *can lead to major clinical problems, which are usually associated with common causes of catheter-associated bacteriuria, infections of the urinary and hepatobiliary tracts [1-5], wound infection, and septicaemia [6-9]. A few devastating infections with *M. morganii *that were associated with a high mortality rate following bacteraemia sepsis and/or nosocomial infection have also been reported, although most of such infections respond well to appropriate antibiotic therapy [3,10-13].

Although *M. morganii *was formerly classified as *Proteus morganii *[14], it was later assigned to the genus *Morganella *on the basis of DNA-DNA hybridisation results [15]. Members of the genus can ferment trehalose, and express lysine decarboxylase and ornithine decarboxylase [16].

Like other members of the *Enterobacteriaceae, M. morganii *has a natural resistance to β-lactam antibiotics [17]. Many strains of *M. morganii *are resistant to the drugs cefazolin, cefixime, cefpodoxime, and ampicillin [1,2,18,19]. Members of the tribe *Proteeae*, which include *Proteus, Providencia *and *M*. *morganii *share homologous genes acquired from horizontal gene transfer via conjugative integration or mobile transposition [20-25]. The drug resistance of *M*. *morganii *was introduced via extra genetic elements [26,27] and/or mobile elements [23,24]. The resistant strains that carry *bla*_CTX-M _gene are capable of producing β-lactamases [28], which can break down the extended spectrum β-lactam drugs [29].

Complicated urinary tract infections, especially those associated with long-term catheterisation may be caused by polymicrobes, as well as biofilm formation. In addition to *M. morganii, Escherichia coli, P. mirabilis, Providencia stuartii, Klebsiella pneumoniae*, and *Pseudomonas aeruginosa *frequently cause urinary tract infections [2,30,31]. Like *P. mirabilis, M. morganii *is motile, with peritrichous flagellar. The flagella-encoding genes are located in a contiguous manner in a single locus of the *P. mirabilis *genome [32]. Besides flagella, adherence is another major determinant of bacterial colonisation and biofilm formation. Several fimbriae have also been shown to play important roles in establishing complicated urinary tract infections [33-37]. They are type-1 fimbriae, mannose-resistant/*Proteus*-like (MR/P) fimbriae, uroepithelial cell adhesin (UCA; also called NAF for nonagglutinating fimbriae), type-3 fimbriae, and *P. mirabilis *fimbriae (PMF; also called MR/K).

The production of urease has a fitness factor that influences bacterial growth and biofilm formation during urinary tract infections. Other virulence factors may include iron acquisition systems, type-3 secretion system (T3SS), two-component systems (TCS), proteins that function in immune evasion (IgA protease), and haemolysins [35].

The environment found in the guts of nematodes or insects may be an important determinant of bacterial pathogenicity [38]. Ethanolamine, which is abundant in human diets and the intestinal tracts of humans, can be used by gut bacteria as a source of carbon and/or nitrogen [39]. The association between the use of ethanolamine and the virulence of various pathogens has been reported [39].

Phylogenetic assessment of 16S rRNA sequences indicates that *P. mirabilis *is the closest relative of *M. morganii*. Only one complete *Protues *genome sequence and four draft sequences of *Providencia *spp. are available.

Here we report the draft genome sequence of a clinical isolate of *M. morganii *and the results of its bioinformatic analysis to enhance understanding of *M. morganii *biology. Comparative analysis of the sequences with the sequences of other *Proteeae *and *Enterobacteriaceae *genomes identifies potential virulence determinants, which may provide new drug targets.

## Results

### Epidemiological study of *M. morganii *infection

Over a 6-year period (2006-2011), samples were collected from all patients at Changhua Christian Hospital, Taiwan, who presented with symptoms of Gram-negative bacterial infections. Of 82,861 samples, 1,219 (1.47%) were positive for M. *morganii *and 3,503 (4.23%) were positive for *Proteus *spp. As shown in Table 1, *M. morganii *was ranked between the eighth and fourteenth most prevalent Gram-negative bacterial species isolated from the hospital over 12 consecutive 6-month intervals during the 6 years of the study.

**Table 1:** The Changhua Christian Hospital annual infections report (2006-2011)

Gram (-) Bacterium/Rank	2006 (1-6)	2006 (7-12)	2007 (1-6)	2007 (7-12)	2008 (1-6)	2008 (7-12)	2009 (1-6)	2009 (7-12)	2010 (1-6)	2010 (7-12)	2011 (1-6)	2011 (7-12)	# infections	Rank
*Escherichia coli*	1	1	1	1	1	1	1	1	1	1	1	1	24,698	1
*Pseudomonas aeruginosa*	2	2	2	2	2	2	2	2	2	3	4	3	13,390	2
*Klebsiella pneumoniae*	3	3	3	3	3	3	3	3	3	2	3	2	10,902	3
*Haemophilus influenzae*	4	5	4	5	4	4	4	4	4	5	2	5	7,124	4
*Acinetobacter baumannii complex*	5	4	5	4	5	5	5	7	5	4	5	4	4,927	5
*Proteus mirabilis*	7	6	6	6	7	6	7	5	6	6	6	6	3,324	6
*Enterobacter cloacae*	6	7	7	7	8	8	8	6	7	7	7	7	2,794	7
*Stenotrophomonas maltophilia*	8	10	10	10	11	10	12	12	9	9	9	10	1,538	8
***Morganella morganii***	**12**	**11**	**14**	**13**	**10**	**12**	**13**	**17**	**12**	**8**	**10**	**13**	**1,219**	**9**
*Serratia marcescens*	11	14	13	16	14	16	14	13	11	13	11	12	1,061	10
*Salmonella group B*	15	15	19	15	18	13	20	11	17	12	17	9	873	11
*Salmonella enteritidis group D*	21	19	20	18	NA	18	15	15	13	11	13	11	706	12

The frequency of infection by 21 bacterial species was monitored over 6-month intervals (the first and second halves of each year) and ranked such that 1 denotes the species most frequently associated with bacterial infection. The column heading '# infections' represents the total number of patients infected with the species indicated. NA: data not available, not significant to the ranking list.

The KT strain of *M. morganii *was isolated from the blood of a patient who developed sepsis during postoperative care. The KT strain was found to be susceptible to amikacin, ertapenem, gentamicin, meropenem, and cefepime but resistant to ampicillin, amoxicillin-clavulanate, cefazolin, cefuroxime, cefmetazole, flomoxef, and cefotaxime.

### General features of the *M. morganii *draft genome

The genome of the *M. morganii *strain KT, which carries no plasmids, was assembled *de novo *into 58 contigs (each >200 bp long), which together comprised 3,826,919 bp with a GC content of 51.15%. The largest contig is 410,849 bp long, and the N50 statistic (the minimum contig length of at least 50% of the contigs) is 240,446 bp, with pair-end short read sequencing coverage **>1,150-fold**. Seven small contigs (each <13 kb) had low-depth reads (**0.36- to 0.66-fold**), whereas two pairs and one triad shared high sequence identity with minor differences at their ends (Additional file 1). The origin of replication assigned on the basis of the GC-skew analysis together with the location of the *dna*A gene and DnaA boxes of the genome lies between *gidA *(MM01685) and *mioC *(MM01686) (Additional File 1: Figure S1).

As shown in Table 2, comparison of the *M. morganii *sequences with the complete genome of *P*. *mirabilis *HI4320 revealed a 12.2% difference in GC-content of the two species (51.1% for *M. morganii *vs. 38.9% for *P*. *mirabilis*).

**Table 2:** Genome analysis of *M. morganii *KT and P. *mirabilis *HI4320

Feature	*Morganella morganii *KT contigs	*Proteus mirabilis *HI4320 chromosome
**Genome**	58	None
Contig numbers	240,446	None
N50 contig size (bp)	3,826,919	4,063,606
Total contig length (bp)	51.15	38.9
**Protein-coding genes**		
No. of predicted genes	3,565	3,607
CDS with assigned to COGs	2,870	2,850
Number of Transposases	21	33
(IS3, IS4, IS10R)		
**Non-protein-coding genes**		
tRNAs	72	83
23S rRNA	1(~7.9X)	7
16S rRNA	1(~8.2X)	7
5S rRNA	8	8

The draft genome of *M. morganii *strain KT were *de novo *assembled into 58 contigs (length > 200 bp), the N50 contig size is 240,446 bp, (N50: the minimum contig length of at least 50% of the contigs), which together comprised 3,826,919 bp with GC content of 51.15%. *P. mirabilis *HI4320 chromosome has total 4,063,606 bp and G+C content of 38.9% [32].

Database searches identified 3,565 predicted coding sequences (CDSs). Among them, 2,870 CDSs could be placed into clusters of orthologous groups with assigned biological functions, and 21 transposes have also predicted. As *P. mirabilis *identified 3,607 predicted CDSs, 2,850 could placed into clusters of orthologous groups and have 33 transposes predicted.

*M. morganii *have predicted 72 tRNAs, as *P. mirabilis *have 83 tRNAs. *M. morganii *have one 16S rRNA, one 23 SrRNA, and eight 5S rRNA were predicted, Further analysis of contigs revealed that the 16S rRNA had a read depth of 7.9-fold and that the 23S rRNA had a read depth of 8.2-fold.

Sequences that encode eight 5S rRNAs, one 16S rRNA, and one 23S rRNA were identified using the ribosomal RNA scan application in RNAmmer (http://www.cbs.dtu.dk/services/RNAmmer/) (Table 2). Further analysis of contigs revealed that the 16S rRNA had a read depth of 7.9-fold and that the 23S rRNA had a read depth of 8.2-fold (Additional file 2: Supplementary table 1).

### Coding DNA sequences

**Figure 1:**
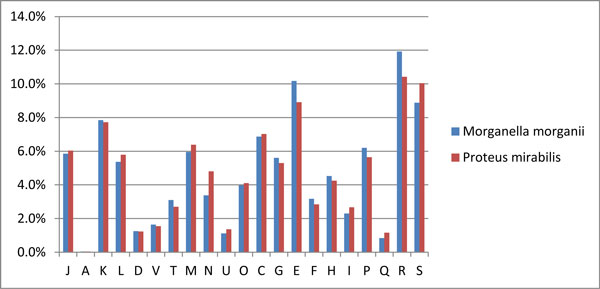
**Compare functional category of *in silico *predicted proteins**. Functional category of in silico predicted proteins. The colors used in the bars represent different object: blue, *Morganella morganii *KT; red, *Proteus mirabilis *HI4320. Proteins were clustered by COG assignment: [1] Information storage and processing: (J) translation, ribosomal structure and biogenesis; (A) RNA processing and modification; (K) transcription; (L) replication, recombination and repair; [2] Cellular processes and signalling: (D) cell cycle control, cell division, chromosome partitioning; (V) defense mechanisms; (T) signal transduction mechanisms; (M) cell wall/membrane/envelope biogenesis; (N) cell motility; (U) intracellular trafficking, secretion, and vesicular transport; (O) posttranslational modification, protein turnover, chaperones; [3] Metabolism: (C) energy production and conversion; (G) carbohydrate transport and metabolism; (E) amino acid transport and metabolism; (F) nucleotide transport and metabolism; (H) coenzyme transport and metabolism; (I) lipid transport and metabolism; (P) inorganic ion transport and metabolism; (Q) secondary metabolites biosynthesis, transport and catabolism; [4] Poor characterized: (R) general function prediction only; (S) function unknown.

Database searches identified 3,565 predicted coding sequences (CDSs). Among them, 2,870 CDSs could be placed into clusters of orthologous groups with assigned biological functions (Figure 1). The proteins annotated as pathogenicity and fitness factors are listed in Table 3, with additional information in Tables 4, 5, 6 and Additional Files 3, 4, 5, 6, 7, 8: Supplementary tables 2-7.

**Table 3:** Pathogenicity and fitness factors identified from analysis of the genome of *M. morganii *KT

Category	Niche genes of *M. morganii *KT
Drug resistance	Ampicillin resistance, *ampC-ampR*. Metallo-β-lactamases (MBL) MM2254, MM2308, and MM2606. Tellurite resistance operon MM933-1004. Tetracycline resistant, chloramphenicol, acetyltransferase, bicyclomycin, acetyltransferase, kasugamycin *tetAJ, catA2, bcr*, catB3-like, *ksgA*.
	
	Multidrug resistance and efflux genes (34 genes, Additional File 3: Supplementary table 2).

Fimbrial adhesins	3 MR/P operons, 13 mrpJ paralogous, 1 fimbrial chaperone, 2 UCA (NAF) operons, 1 PMF operon, and 2 other operons (Tables 4, 5).
	
	6 putative type IV pili genes *hofCB, ppdABCD*, and 2 putative trimeric autotransporter secretion genes MM2011, MM2042.

Motility/flagellum-related	Most flagellum-related genes and chemotaxis genes; MM1735-1785, MM1796-1797, and MM1786-1793 (at a single locus) (Additional File 4: Supplementary table 3).
	
	Methyl-accepting chemotaxis gene MM0264, aerotaxis gene MM1607, and 9 regulators of flagellar genes *umoABCD, rssBA, rcsBCD*.

T3SS	Type III secretion system needle complex MM0224-MM0243 (20 genes), and effectors, *ipaCBD*-chaperone (MM0244-MM0247, 4 genes) (Additional File 5: Supplementary table 4)

TCS	19 potential two-component regulator systems (TCS) were identified.

Iron acquisition system	Heme uptake, ferric, ferrous iron transport, ferric siderophore receptor, B_12 _transporter, and siderophore iron uptake *hmuSTUV, afuABC, feoAB, ireA, btuCD, btuB*, and *yfeDCBA*
	
	18 other related genes (*fecR*, ABC transporters, TonB-dep. receptors)

IgA protease	Zinc metalloprotease, capable of cleaving host Ig, *zapABCD *(MM1054-MM1056, MM1058)

LPS, ECA & capsule	(1) Release lipid A, induce initial endotoxic shock and enterobacterial common antigen (41 genes, Additional File 6: Supplementary table 5) (2) Protect from phagocytosis, *rcsB,C,D,F*

Haemolysins	*hmpBA *(MM2452, MM2453)

Ureases	Urea as a nitrogen source, rapid urea hydrolysis, a cause of stone formation. *ureABCEFGD*-urea transporter (MM1961-MM1968)

Insecticidal& apoptotic toxins	9 insecticidal toxin: (1) *tccB, tccA*, and *tcdB*2 (MM0965-MM0967), (2) *xptA1A1C1 *(MM1780-1782), (3) *tcaC, tccB*3, and *tcaC *(MM1901, MM2567 and MM2570) (Additional File 7: Supplementary table 6)
	
	RtxA, XaxAB, intimin/invasion, HlyD toxin secretion, toxin transporter

Other genes required for persistence of infection and fitness for infection	6 superoxide stress genes *katA, soxS, sodC,B,A, oxyR *(Additional File 8: Supplementary table 7) The phage-shock-protein, *pspABC *operon (MM0627-MM0629)

Ethanolamine utilisation	Ethanolamine utilisation system, provide carbon and/or nitrogen uptake; *eutSPQTDMNEJGHABCLK*-*pduST*-*eutR *(MM1148-MM1130). (Table 6)

Cobalamin biosynthesis	Cobalamin (vitamin B_12_) biosynthesis, cbi-cob operon MM1167-MM1187.

**Table 4:** Chaperone-usher fimbrial operons

Genes	Designation
MM0177-MM0183	*mrp''*
MM0279-MM0281	*uca'*
MM0293-MM0300	*mrp*(mannose-resistant/Proteus-like fimbria)
MM0304-MM0312	*mrp'*
MM0959-MM0962	Fimbria 5
MM2683-MM2686	*pmf *(*P. mirabilis *fimbria); also called MR/K
MM2740-MM2745	*uca *(uroepithelial cell adhesin); also called naf (nonagglutinating fimbria
MM2839-MM2842	Fimbria 8

**Table 5:** mrpJ paralogues in *M. morganii*

Name	Locus	Fimbrial operon
*mrpJ*	MM0301	MR/P
*mrpJ'*	MM0314	MR/P'
MM0158	MM0158	Orphan
MM0206	MM0206	Orphan
MM0209	MM0209	Orphan
MM0323	MM0323	Orphan
MM0639	MM0639	Orphan
MM0640	MM0640	Orphan
MM2529	MM2529	Orphan
MM2705	MM2705	Orphan
MM2712	MM2712	Orphan
MM2969	MM2969	Orphan
MM2970	MM2970	Orphan

**Table 6:** Protein similarity search of ethanolamine utilization (*eut*) operon of M. morganii

*eut *genes			
*eutS*	Eco (85%)	Eb(84%)	Ko(84%)
*eutP*	Eco (67%)	Ss(66%)	Sb(66%)
*eutQ*	Se (63%)	Ko(63%)	Yi(63%)
*eutT*	Eco (65%)	Ko(65%)	Yi(64%)
*eutD*	Eco (72%)	Ko(70%)	Yi(69%)
*eutM*	Yi (96%)	Eco(94%)	Ecl(94%)
*eutN*	Yi (84%)	Cy(74%)	Ecl(74%)
*eutE*	Ck (78%)	Eco(77%)	Yi(76%)
*eutJ*	Yi (73%)	Eco(71%)	Cy(70%)
*eutG*	Yi (67%)	Ko(59%)	Se(58%)
*eutH*	Yi (82%)	Cy(80%)	Yr(80%)
*eutA*	Yi (76%)	Pa(70%)	Cy(67%)
*eutB*	Yi (85%)	Cy(85%)	Yr(85%)
*eutC*	Eco (78%)	Ko(77%)	Ecl(77%)
*eutL*	Cy (87%)	Se(85%)	Ecl(85%)
*eutK*	Eco (56%)	Se(53%)	Cy(51%)
*pduS*	Ha (66%)	Yi(66%)	Eb(65%)
*pduT*	Ta (63%)	Yi(63%)	Eb(63%)
*eutR*	Yi (62%)	Ko(56%)	Eco(56%)

*M. morganii *has longest ethanolamine utilization (*eut*) operon, *eutSPQTDMNEJGHABCLK-pduST-eutR *which consists of 19 genes.

The two novel genes in *eut *of *M. morganii *KT, *pduT *and *pduS*, were found orthologous protein from *pdu *operon from other species. Sequence identity of best top three hits ranging from 51 to 87% and species were varied. Abbreviation of species were Eco: *Escherichia coli*; Eb: *Enterobacteriaceae bacterium*; Ko: *Klebsiella oxytoca*; Ss: *Shigella sonnei*; Sb: *Shigella boydii*; Se: *Salmonella enterica*; Yi: *Yerisinia intermedia*; Yr: *Yokenella regensburgei*; Ecl: *Enterobacter cloacae*; Cy: *Citrobacter youngae*; Ck: *Citrobacter koseri*; Ta: *Tolumonas auensis*; Ha: *Hafnia alvei*; Pa: *Photorhabdus asymbiotica*. Id (%) represent percent of identify compare two orthologous proteins.

### Prophages and mobile elements

Bacteriophages and transposons, which may contribute to genome plasticity [40], are, in general, laterally acquired elements [32]. In the *M. morganii *genome, 2 prophages (MM3229 through MM3271 and MM2276 through MM2290) and 12 degenerate prophages together comprise 236 prophage-related genes (Additional file 9: Supplementary table 8). The prophage 1 genes were also found in the genome of *Proteus mirabilis, Providencia alcalifaciens*, and *Providencia rustigianii*, whereas the prophage 2 genes are orthologous to those found in the other 14 *Enterobacteriaceae *genomes.

Neither the integrated conjugative element ICE*Pm*1 (reported in certain *M. morganii *strains and *P. mirabilis *[30]) nor ICE/R391 (reported in *Providencia *spp. and *P. mirabilis *[24]) was found in *M. morganii *strain KT. However, several integrase recognition sequences, including *prf*C (MM1941) and two tRNA PheV sites (contig 1: 376,829 - 376,904 bp and contig 12: 37,047-37,122 bp), are present in the strain KT genome.

### Drug resistance

A chromosomally encoded β-lactamase from *M. morganii *has been cloned [41]. The clinical strains with high or low levels of cephalosporinase expression were found to harbour the adjacent *ampC *(MM3167) and *ampR *(MM3166) genes, which are flanked by the *hybF *and *orf-1 *genes [17]. The *ampC *and *ampR *genes produce both broad-spectrum and the *ampC *β-lactamases or only the *ampC *β-lactamase [42]. In *Providencia *spp., the production of metallo-β-lactamases has been associated with resistance to carbapenems [43,44]. The *M. morganii *KT strain also contains the gene encoding β-lactamase, which confers resistance to aminopenicillins. Genes that encode three metallo-β-lactamases (MM2254, MM2308, and MM2606) were found. Moreover, the *M. morganii *genome also encodes the tetracycline resistant protein TetAJ (MM3521) and the chloramphenicol acetyltransferase CatA2 (MM3053).

Other drug resistance genes include a tellurite resistance operon (MM0993 through MM1004), the bicyclomycin (sulphonamide) resistance gene *bcr *(MM2780), a *catB3*-like putative acetyltransferase gene (MM0469), and a kasugamycin resistance gene *ksgA *(MM2040). The tellurite resistance *ter *operon was also identified in *P. mirabilis *HI4320, *P. stuartii *ATCC# 25827, *K. pneumoniae *NTUH K2044, *E. coli *O157:H7, and *E. coli *APEC O1. The genes involved in multidrug efflux and the CDSs predicted to confer antimicrobial resistance are listed in Additional file 10: Supplementary table 9.

### Fimbriae, pili, and adhesion proteins

**Figure 2:**
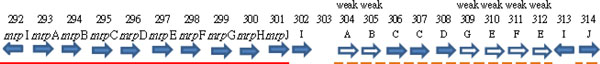
**The duplicated MR/P fimbrial operons of *M. morganii*. **The *mrp *operon (*mrpABCDEFGHJ*, solid red line region) is an important determinant of virulence in bacteria responsible for urinary tract infections. *mrpI *encodes a recombinase that controls the invertible promoter element of the *mrp *operon. A duplicated operon *mrp' *(*mrpABCCDGEFE*, broken orange line) is located immediately downstream of ***mrpJ***. The numbers 292 through 314 represent the identifiers of *M. morganii *KT gene products MM0292 through MM0314. The term 'weak' denotes limited homology to the paralogous pair in *mrp*.

As shown in Table 4, eight potential chaperone-usher fimbrial operons were identified. Among them, three were previously identified as the operons that encode the components of the MR/P, UCA (NAF), and PMF (MR/K) fimbriae [33,34]. An apparent duplicate of the *mrp *operon immediately adjacent to *mrp*J was designated *mrp' *(Figure 2). This is similar to the duplicated operon present in *P. mirabilis*, albeit with different annotation and orientation of the constituent genes [32]. Interestingly, the *M. morganii *genome contains 13 paralogues of *mrpJ*, which encodes a transcriptional regulator that represses motility [45]. As shown in Table 5, two *mrpJ *genes were associated with each of the fimbrial operons.

Together with the *hofC *(MM3190) and *hofB *(MM3191) genes, four genes, namely *ppdA *(MM2057), *ppdB *(MM2058), *ppdC *(MM2060), and *ppdD *(MM3192), are involved in the assembly of type-IV pili. Two genes that encode putative trimeric autotransporter adhesins (MM2011 and MM2242) are homologues of proteins found in *P. stuartii *ATCC# 25827 and *Y. enterocolitica *8081.

### Motility and chemotaxis systems

Fifty three genes that encode proteins required for flagellar structure (MM1735 through MM1785, as well as MM1796 and MM1797) and eight chemotaxis-related genes (MM1786 through MM1793, including *cheA, cheW, cheD, tap, cheR, cheB, cheY*, and *cheZ*) are contained in the *M. morganii *genome. With the exception of the genes that encode two LysR family transcriptional regulators (MM1739 and 1765), the camphor resistance protein CrcB and related genes (MM1743 through MM1749), a short-chain dehydrogenase/reductase SDR (MM1764), and the three insecticidal toxin complex proteins encoded by *xptA1A1C1 *(MM1780 through MM1782), the organisation of flagellar genes is similar to that of *P. mirabilis*.

The genes that flank the locus that encodes flagellar structural proteins encode a methyl-accepting chemotaxis protein (MM0264) and an aerotaxis protein (MM1607). Genes that encode regulators of flagellar motility, such as *umoA *(MM1647), *umoB *(MM3381), *umoC *(MM1924), *umoD *(MM2830), *rssBA *(MM2256 and MM2257), and the *rcsBCD *phosphorelay regulatory system (MM1705 through MM1707), were also identified.

### Iron acquisition

The *M. morganii *genome encodes the *hmuSTUV *haeme uptake system (MM0575 through MM0578), the *afuABC *system for ferric and ferrous iron transport (MM1068 through MM1070), *feoAB *(MM2728 and MM2729), the ferric siderophore receptor locus *ireA *(MM3404), the B_12 _transporter locus *btuCD *(MM0560 and MM0561), *btuB *(MM2756), and the siderophore iron uptake systems *yfeDCBA *(MM0546 through MM0549) to circumvent host iron-sequestering mechanisms. A possible haeme-binding system is encoded by MM1650 and MM1651. A gene that encodes a putative iron receptor (MM1952) is located adjacent to a *fecR *homologue (MM1953). There are two other iron-related ABC transporters (encoded by MM2795 and MM2796, and MM0258 through MM0260) and 12 additional TonB-dependent receptors (encoded by MM0169, MM0255, MM1275, MM1299, MM1650, MM1872, MM1952, MM2348, MM2405, MM2430, MM2542, and MM2798).

### Zinc acquisition

The *znuACB *high-affinity zinc transporter system was recently shown to be a fitness factor for *E. coli *and *P. mirabilis *during experimental urinary tract infection [46,47]. The system is encoded by *M. morganii *gene products MM2146 through MM2148.

### Type III secretion system

A T3SS, which comprises gene products MM0224 through MM0247 and has a low GC-content (43.7%), resides in a 20.8-kb pathogenicity island. This island of 24 genes, which shares homologous syntenic blocks with *P. mirabilis *[48], contains all the components needed to assemble a T3SS needle complex. Except for the genes that encode effector proteins, the most homologous proteins were orthologues from *P. mirabilis *(Additional File 5: Supplementary table 4). A putative *IpaCBD *operon, which encodes chaperones for three proteins (MM0244 through MM0247) was also found. The IpaC and IpaD proteins have low similarity (21% and 38%, respectively) to those of *P. mirabilis*, whereas the IpaB protein has the highest homology with ipaB of *P. mirabilis *but low similarity (20-30%) with that of *Shigella *spp. and *Salmonella enterica *subsp.

### Two-component systems

Nineteen potential TCS were identified. These include the quorum sensors *qseBC *(MM2889, MM2890), *yedWV *(MM2314, MM2315), and *BarA/UhpA *of the *LuxR *family (MM3087, MM1090). Nineteen orthologues that encode TCS components were identified in *P. mirabilis *HI4320 [32], *P*. *alcalifaciens *NCTC 10286; r04, *P. rettgeri *DSM# 1131, *P. rustigianii *DSM# 4541, *Enterobacter aerogenes *KCTC 2190, *Photorhabdus luminescens *laumondii TTO1, and *Y. enterocolitica *8081.

### Regulation

Analysis of the *M. morganii *genome identified seven sigma factor subunits of RNA polymerase. These are the major sigma factor *rpoD *(σ^70^,σ^A^) encoded by MM1372, and six alternative sigma factors: *rpoH *(σ^32^, σ^H^) encoded by MM1254, *rpoN *(σ^54^, σ^N^) encoded by MM1306 to control promoters for nitrogen assimilation, *rpoS *(σ^38^, σ^S^) encoded by MM1208 to activate stationary-phase promoters, *rpoE *(σ^24^, σ^E^) encoded by MM1906 to regulate extra-cytoplasmic stresses, *rpoF *(σ^28^, σ^F^) encoded by MM1736 to regulate flagellum-related functions, and a FecR family sigma factor encoded by MM1954.

The small RNA regulatory gene *ryhB *found in *E. coli *and *P. mirabilis *[49], which regulates a set of iron-storage and iron-usage proteins, is present in only a single copy (contig 25).

### IgA protease

The *zapA *gene encodes a zinc metalloprotease that cleaves a broad range of host proteins, including serum and secretory IgA1, IgA2 and IgG, complement proteins, and antimicrobial peptides [35]. Whereas the *M. morganii *KT, *P. mirabilis *HI4320, and *P. luminescens *laumondii TTO1 genomes all encode *zapABCD *(MM1054-MM1056, MM1058), the *P. mirabilis *HI4320 genome encodes *zapEEEABCD *[32].

### Lipopolysaccharide and the cell capsule

Lipopolysaccharide (LPS), the main structural component of the outer membranes of Gram-negative bacteria, consists of a lipid A molecule and a variable O-antigen. Lipid A released during bacterial lysis induces endotoxic shock. Several O-specific polysaccharides of *M. morganii *have been investigated [50,51], and at least 55 O-antigens have been identified [52]. The genes predicted to be involved in the synthesis of LPS and the enterobacterial common antigen [53] are listed in Additional File 6: Supplementary table 5. Four genes, *rcsB *(MM1706), *rcsC *(MM1707), *rcsD *(MM1705), and *rcsF *(MM2111), are implicated in the regulation of capsule synthesis.

### Immunity-like system

Clusters of regularly interspaced short palindromic repeats consist of multiple short nucleotide repeats, which are separated by unique spacer sequences flanked by characteristic sets of CRISPR-associated genes [54,55]. The CRISPR-associated proteins MM3304, MM3306, and MM3307 were identified downstream of the degenerate prophage #6. Comparison of the *M. morganii *genome with that of the other 14 members of the *Enterobacteriaceae *analysed revealed orthologues in the pathogens *P. stuartii *ATCC# 25827, *E. coli UTI89*, and *E. coli APEC O1*, all of which have been implicated in causing urinary tract infections [56].

### Haemolysins

The gene *hmpA*, encoding a secreted haemolysin, was originally identified in uropathogenic isolates [57,58]. The two partner genes, *hmpBA*, encoding secreted proteins that are highly conserved in *P. mirabilis *[59], are encoded by MM2452 and MM2453 in *M. morganii *KT. Functional similarity of the *E. coli *and *M. morganii *homologues of HmpBA was reported previously [60].

### Urease hydrolysis and putrescine production

Rapid urea hydrolysis is a prominent phenotype of *Proteeae *organisms [61]. The urease enzyme is believed to be a cause of urinary stone formation [62]. The urease of *M. morganii*, which revealed a high degree of amino acid conservation to *P. mirabilis *urease, has been purified and characterised [4]. Ureases from both *M. morganii *and *P. mirabilis *urease gene cluster are required for virulence [63]. The analysis revealed that the closest homologues of the members of the urease gene cluster *ureABCEFGD *were the orthologues from *Yersinia pseudotuberculosis *and *Y. enterocolitica*, with amino acid identities that ranged from 69% to 94%. Unlike the urease gene cluster of *P. mirabilis*, the *M. morganii *genome has a gene that encodes the transporter MM1968, in addition to MM1961 to MM1967. On the other hand, the *P. mirabilis ureR *gene, which encodes a transcriptional activator, was not found in the genome.

The genome of *M. morganii *KT contains genes for two pathways involved in putrescine production. The first of these pathways involves ornithine decarboxylase, which is encoded by *speF *(MM3013), and the putrescine transporter, which is encoded by *potE *(MM3012). The enzymes in the second pathway, which are encoded by *carAB *(MM2009 and MM2010), *argI *(MM0127), *argG *(MM1552), *argH *(MM1551), *speA *(MM2553), and *speB *(MM2554), also participate in urea production. All of the urease cluster genes that encode enzymes from both pathways have orthologues in *P. mirabili*s HI4320 [32].

### Toxins

Several CDSs that encode potential toxins were found. These include a gene that encodes the cytotoxin RtxA (MM0676), the two *XaxAB *genes (MM0454 and MM0455) that encode apoptotic toxins, a putative intimin gene for host-cell invasion (MM0208), a HlyD-family toxin scretion protein (MM2481), and a transporter gene (MM2482). Among the nine insecticidal toxin-related genes (Additional File 7: Supplementary table 6), *tccB, tccA*, and *tcdB*2 (MM0965 through MM0967) are orthologous to the insecticidal toxin genes of *Pseudomonas *spp. The three other insecticidal toxin genes *xptA1A1C1 *(MM1780 through MM1782) were identified in a continuous locus between the flagellum-related genes and chemotaxis genes.

### Ethanolamine utilisation system

The ethanolamine utilisation system, which is encoded by the *eut *operon it were found to vary substantially between species [39], is composed of the genes *eutSPQTDMNEJGHABCLK*-*pduST*-*eutR *(MM1148 through MM1130). This region carries two extra genes compared to the 17 *eut *genes found in other *Enterobacteriaceae *genomes [64]. In addition, the gene organisation is unique, with *pduST *located between *eutK *and *eutR*. As shown in Table 6, the sequence similarities of the three most similar proteins ranged from 51% to 87%, and species varied.

The comparison revealed that the 17-gene operon is present in *Klebsiella pneumonia, E. coli*, and *Salmonella enterica *serovars. The *pdu *operon, a paralogous operon required for use of propanediol that is found in these three species, was not found in the *M. morganii *KT genome.

Located upstream of the *eut *operon, MM1168 through MM1187 encode the enzymes of the *cob-cbi *operon, which is required for cobalamin (vitamin B_12_) biosynthesis [65-67]. Under aerobic conditions, the activity of EutBC depends on the exogenous supply of cobalamin.

### Other determinants of persistence of infection and fitness for infection

Pathogenic bacteria produce superoxide dismutase to protect them from being killed by the reactive oxygen species generated by their hosts [68]. As shown in Additional File 8: Supplementary table 7, the genes involved in countering superoxide stress are *katA, soxS, sodC, sodB, oxyR*, and *sodA*.

The *pspABC *operon (MM0627 through MM0629) encodes a phage-shock-protein (*psp*), which ensures the survival of *E. coli *during late-stationary-phase stresses [69-71].

### Prominent virulence and fitness factors in *M. morganii *KT--a comparison with *Enterobacteriaceae *and *Proteeae *genomes

Sequence comparison between *M. morganii *KT and the 14 members of the *Enterobacteriaceae *family, including 5 *Proteeae *species (*P. mirabilis *HI4320, *Providencia stuartii *ATCC# 25827, *P. alcalifaciens *NCTC 10286, *P. rettgeri *DSM# 1131, and *P. rustigianii *DSM# 4541), 4 *E. coli *strains (O157:H7 EC4115, K-12, UTI89, and APEC O1), *K. pneumonia *NTUH K2044, *Enterobacter aerogenes *KCTC 2190, *Photorhabdus luminescens *laumondii TTO1, *S. enterica *serovars Typhimurium LT2, and *Y. enterocolitica *8081, revealed that 459 CDSs found in *M. morganii *are not found in the other *Proteeae *species studied, and 295 CDSs found in *M. morganii *are not found in any of the 14 *Enterobacteriaceae *genomes studied (Additional file 10: Supplementary table 9). The genes specific to *M. morganii *include the genes in the *eut *operon, *cob-cbi *operon, 8 insecticidal toxin genes, 9 T3SS genes, and 17 copies of the IS4 family transposase gene.

Among the orthologous genes, 2,411 CDSs were found in the genomes of the 5 *Proteeae *species analysed, and 1,920 CDSs were found in the 14 *Enterobacteriaceae *genomes analysed. As shown in Additional file 11: Supplementary table 10, of the genomes analysed, that of *P. rettgeri *is the most closely related genome to that of *M. morganii*, sharing 2,802 orthologous CDSs. The orthologous genes encode proteins for drug resistance, pathogenicity (IgA protease and LPS), motility, iron acquisition, ethanolamine use, and urease production, as well as components of an immunity-like system (CRISPR) and fimbrial adhesins, toxins, and haemolysin.

## Discussion

Epidemiological studies have revealed that *M. morganii *is frequently isolated from clinical specimens collected from patients with nosocomial bacterial infections [3,5,11,72-74]. Strains of *M. morganii *that confirm the chromosomal origin of the plasmid-located cephalosporinases [17] are now found throughout the world [75]. Genes from *M. morganii *that encode cefotaxime-hydrolysing β-lactamases have also been reported with increasing frequency [42]. Plasmid-borne drug resistance factors have also increased the virulence of *M. morganii *[26,27]. Gene products that confer multidrug resistance, including metallo-β-lactamases and efflux pumps for tetracycline, tellurite, bicyclomycin, and kasugamycin, have been commonly reported for clinical isolates of *M. morganii*. In the event of an outbreak, this situation poses a potential threat owing to the absence of a proper antibiotic therapy.

Compared with other members of the genus *Proteeae*, which have GC contents that range from 39% to 43%, the GC content of *M. morganii *is 51%. which is genetically different from other species [76,77] and therefore assigned to the genus *Morganella *[14,15,61,76]. The plasmid-borne gene that encodes lysine decarboxylase was once used to classify bacteria [78,79]. However, the chromosomal gene that encodes ornithine decarboxylase was subsequently adopted as a characteristic feature to classify members of the genus *Morganella *[80,81].

Information on the 16S rRNA gene and paralogs in genome is important for evolution and bacterial population studies [40]. The *P. mirabili*s genome has seven rRNA operons (six 16S-23S-5S operons and one 16S-23S-5S-5S operon) [32]. Analysis of the *M. morganii *KT genome sequence revealed eight duplications of the 16S and 23S rRNA genes while 5S rRNA were also 8. The prophage genes that were found in the other *Enterobacteriaceae *species appeared to comprise 7% of the *M. morganii *genome.

Interestingly, the ICE*Pm*1 and ICE/R391 genes, which are present in many *P. mirabilis *isolates [21], are not found in strain KT. Seventeen copies of transposase genes of the IS4 family were not present in other *Proteeae *genomes, which implies that different transposition events occurred during the evolution of *M. morganii *and of these species.

Unlike the flagellar genes of *P. mirabilis, M. morganii *KT has LysR family transcriptional regulatory genes (MM1739 and MM1765), genes MM1743 through MM1749 that encode the camphor resistance gene CrcB and related proteins, a short-chain dehydrogenase/reductase SDR gene (MM1764), and the insecticidal toxin complex genes *xptA1A1C1 *(MM1780 through MM1782).

Both *M. morganii *KT and *P. mirabilis *have duplicated MR/P fimbrial operons, albeit with different numbers of genes [32]. As shown in Figure 2, whereas three copies of the MrpI recombinase gene are found in *M. morganii *KT, only one copy is in the *P. mirabilis *genome. In *M. morganii *KT, the *mrp' *operon is oriented opposite to *mrpI*, whereas *mrpI *is not present in the *P. mirabilis mrp' *operon.

More than half of the pathogenicity island genes are conserved and found to show collinear synteny between *M. morganii *and *P. mirabilis*. However, genes that encode components of T3SSs have low sequence similarities.

EDTA-sensitive protease Zap gene clusters have been found in many *Proteus *spp. and *E. coli *clinical strains but are not produced by *Providencia *spp. and *Morganella *spp. [37]. The *zapABCD *genes found in *M. morganii *KT differ from those in *P. mirabilis *HI4320 *zapEEEABCD *[32] insofar as KT has an incomplete *zapE *gene downstream of *zapABCD*. Although the *M. morganii *urease shares a high degree of sequence similarity to *P. mirabilis *ureases, the presence of the unique transporter gene (MM1968) and the absence of *ureR *in *M. morganii *suggest that the two species use different systems to regulate urease transport.

Although none of the insecticidal toxin genes were found in *P. mirabilis*, some were found in *Xenorhabdus *[82,83], *Pseudomonas *[84], *Yersinia*, and *Photorhabdus *[85]. In *Photorhabdus*, the related toxin complex is released upon invasion of the nematode host [86]. Why the *M. morganii *clinical isolate harbours eight insecticidal toxin genes remains to be investigated.

The urinary and hepatobiliary tracts are two major portals of entry for *M. morganii *[3]. Compared with *P. mirabilis *HI4320 [32], *M. morganii *has fewer types of fimbriae (three vs. five) and fewer gene clusters (8 vs. 17). This implies that *M. morganii *may be less virulent than *P. mirabilis *in the context of urinary tract infections.

Given that the intestine is thought to provide a rich source of ethanolamine [39], the *eut *operon likely plays a critical role in enabling *M. morganii, E. coli, Klebsiella *spp., and *Yersinia *spp. to use ethanolamine as a source of carbon and/or nitrogen to colonise the intestine. However, the other *Proteeae *genomes lack the *eut *operon and *pduTS*, which together encode proteins that help to establish a microcompartment [87], and the *cob-cbi *operon, which encodes the enzymes needed for cobalamin biosynthesis.

The *eut *operon, which includes *pduST*, and the *cob-cbi *operon likely provide the fitness factors required for colonisation by intestinal bacteria. This may explain why *M. morganii *is more frequently associated with nosocomial bacterial infections than other members of *Proteeae*.

Typically antibiotics target the essential cellular function, like cell-wall synthesis, ribosomal function, or DNA replication [88]. The whole genome sequencing approaches of human hosts and pathogens facilitate the growing understanding of bacteria infectious disease mechanisms and help to reveal crucial host-pathogen interaction sites [89]. The pathogenicity genes allows usage of computation approaches to identify potential drug targets such as the conserved proteins found in common pathogens[90]. T3SSs is highly conserved in many disease-causing gram-negative pathogens and hence has been used as an alternative strategy for drug target design [90,91]. The efflux pumps which help to get rid of toxic substances also promote biofilms, thus making them attractive targets for antibiofilm measure [92,93]. In many bacteria, biofilm formation, siderophore production and adhesion activity are linked traits. Therefore, drugs that could target bacterial adhesions while colonization could be therapeutically useful [94]. Selective toxicity is another antibiotics approach, which aims to have highly effective against the microbial, but no harm to humans. The unique metabolic pathways identified in *Margonella *may be considered as the new drug targets.

### Conclusions

The pathogenicity-related genes identified in the *M. morganii *genome encode drug resistance determinants and factors that influence virulence, such as fimbrial adhesins, flagellar structural proteins, components of the iron acquisition system, T3SS, and TCS, an IgA protease, haemolysins, ureases, and insecticidal and apoptotic toxins. Comparative analysis with 14 other *Enterobacteriaceae *genomes revealed several systems that vary between species. These include transposes of the IS4 family, insecticidal toxins, T3SS components, and proteins required for ethanolamine utilisation and cobalamin biosynthesis. It is interesting to note that neither the *eut *operon (which includes *pduST*) nor the *cob-cbi *operon is found in other *Proteeae *genomes. Nevertheless, the *eut *operon is also found in several other non-*Proteeae *enterobacteria genomes, albeit with different gene organisation.

In summary, this is the first report of an *M. morganii *genome sequence. Comparative genome analysis revealed several pathogenicity-related genes and genes not found in other *Proteeae *members. The presence of the *eut *operon (which includes *pduST*) and the *cob-cbi *operon in *M. morganii *but not in the other *Proteeae *genomes studied may explain why *M. morganii *is more frequently associated with nosocomial bacterial infections. Moreover, the evidence that *M. morganii *shares features with other non-*Proteeae *enterobacteria suggests that horizontal gene transfer has occurred between *M. morganii *and other intestinal bacteria.

## Methods

### Bacterial strains and culture conditions

With approval by the institutional biosafety committee, we isolated *M. morganii *strain KT (year 2009) from blood of a 57-year-old man during postoperative care. The patient had a medical history of type 2 diabetes mellitus, hepatocellular carcinoma, rectal cancer and gallbladder stone. He was admitted for rectal cancer surgery, and received chemotherapy and radiotherapy. Following surgery, bacteraemia caused sepsis.

Colony morphology, Gram staining, oxidase testing (Dry Slide; Difco Laboratories, Detroit, MI), catalase testing, and routine biochemical reactions identified *M. morganii *as the agent responsible for the infection. A presumptive diagnosis of infection with *M. morganii *was confirmed using API-20 kit reagents (Bio Merieux Vitek; Hazelwood, Mo) and the BACTEC NR-860 apparatus (Becton Dickinson Diagnostic Instrument Systems, Franklin Lake, NJ).

### Genomic DNA preparation

We cultured *M. morganii *in trypticase soy broth or on trypticase soy agar. Bacteriological media were purchased from Biostar Inc. (Taiwan).

Genome DNA from the *M. morganii *strain KT was isolated using reagents from QIAGEN DNeasy kits (QIAGEN Inc., Valencia, CA).

### Cloning and sequencing

Genome sequencing was performed using the whole genome shotgun strategy [95]. Genomic DNA sequencing reads of 101 bp pair-end reads, an average distance between pair reads were 200 bp, and were generated using an Illumina GA IIx (Solexa) sequencer [96].

### Draft genome assembly and validation of contigs

Short sequencing reads were assembled into contigs using ABySS version 1.2.7 software [97]. Contigs were evaluated using the re-sequencing program SOAP2 [98], a tool for aligning raw reads into contigs to evaluate and validate the coverage and depths of read information for each contigs. All contigs were aligned with short reads, with a depth threshold of at least 300 reads.

### Identification and annotation of coding sequences and genes encoding tRNAs and rRNAs

The draft genome of *M. morganii *was analysed using our own integrated annotation pipeline composed of prediction and database search tools. Glimmer (version 3.02) was used to predict assembled contig sequences for prokaryotic CDS regions [99]. Potential long CDSs were extracted using the "long-orfs" program from the Glimmer software suite. These long CDSs were then used by Glimmer to predict CDSs in all contigs.

We used BLAST to query non-redundant protein databases with all predicted CDS regions [100] and thereby find and validate significant protein identifications with E value (E < 1e-5). The annotation of validate significant protein with Clusters of Orthologous Groups functional classification were identified from protein genbank records [101]. The EMBOSS analysis package [102] was used to extract and covert sequences and get predicted open reading frame for further manually verified.

We used tRNAscan-SE to predict prokaryotic transfer RNA (tRNA) genes [103]. Ribosomal 5S, 16S, and 23S RNA (rRNA) genes predictions were performed using RNAmmer (version 1.2) [104]. Origins of replication were assigned based of the GC-skew analysis together with the location of the *dna*A gene and DnaA boxes of the genome, using Ori-Finder [105]. Horizontally acquired DNA by anomalies in the G+C content was calculating by perl programming languages.

The previously published genome and protein sequences of *Enterobacteriaceae *genomes were downloaded from the National Center for Biotechnology Information and draft genome and protein sequences (four *Providencia *spp.) of *Enterobacteriaceae *genomes were downloaded from the Genome Institute at Washington University.

We used BLAST program and formatted proteins of other *Enterobacteriaceae *organisms as databases for comparison, validate orthologous protein with E value (E < 1e-4), identity (> 30%) and threshold to length percentage of alignment.

### Confirm of eut operon organisation (at *eutK-pduS-pduT-eutR *order)

PCR was conducted using primer flanking ORFs: *eutK-pduS-pduT-eutR *(MM1133-MM1130) (forward, TAGAGGACAGCCGTGATGTG; reverse, CAAACAGGGTTTCGGTCAGT). PCRs were carried out in 25-μl reactions containing 30 ng of genomic DNA, 1 × buffer, 250μM deoxynucleoside triphosphate, 0.75 μl of *Taq *DNA polymerase, and 0.4 μM concentrations of forward and reverse primers. Reactions were amplified in a thermocycler at 95°C for 2 min, followed by 30 cycles of 95°C for 15 s, 55°C for 30 s, and 72°C for 60 s. Purified PCR products were sequenced (ABI model 3730).

### To assess if the M. morganii strain KT contained plasmids

The plasmid DNA was isolated by the procedure of alkaline denature method [106] and then separated in 1.0% agarose gel by gel electrophoresis. Plasmids presences were subsequently visualised by UV exposure of the ethidium bromide stained gel.

### Nucleotide sequence accession number

The draft genome sequence of *M. morganii *KT has been deposited in the DDBJ/EMBL/GenBank under the accession number ALJX00000000. The version described in this paper is the first version, ALJX01000000.

### Annual hospital infectious information

Information regarding bacteria responsible for clinically relevant infections was collected over a 6-year period for all patients diagnosed with infection by the Infectious Control Committee of Changhua Christian Hospital, a 1,600-bed medical center in central Taiwan. The reports identified bacteria isolated from samples and their sensitivities to the antibiotics amikacin, ampicillin, amoxicillin-clavulanate, cefepime, cefazolin, cefuroxime, cefmetazole/flomoxef, cefotaxime, ertapenem, gentamicin and meropenem.

## Competing interests

The authors declare that they have no competing interests.

## Authors' contributions

THK, YTC, CYT, WCS and HLP helped design the research project. YTC carried out computational work, conducted sequence analysis and interpreted the results, and drafted the manuscript. THK wrote parts of the manuscript related to clinical analyses. WCS, FRH and MFH assisted with assembly work. HLP and FRH assisted with experiments related to biochemistry and molecular biology. CHC, YMT, CEL and HCC isolated, identified, and cultured bacteria. THK, HLP and CYT refined the manuscript. All authors read and approved the final manuscript.

## Supplementary Material

Additional File 1 **Supplementary **Figure 1. The origin of replication was assigned based on the GC deviation of the genome using Ori-Finder (*.pdf) Click here for file

Additional File 2 **Supplementary **table 1. Resequencing analysis on assembled contigs (*.xls) Click here for file

Additional File 3 **Supplementary **table 2. *M. morganii *genes involved in multidrug efflux genes (*.pdf) Click here for file

Additional File 4 **Supplementary **table 3. Flagellum-related genes and chemotaxis genes located in 58.8-kb locus of *M. morganii *(*.pdf) Click here for file

Additional File 5 **Supplementary **table 4. Protein similarity search of Type III secretion system (T3SS) of *M. morganii *(*.pdf) Click here for file

Additional File 6 **Supplementary **table 5. *M. morganii *genes involved in lipopolysaccharide or enterobacterial common antigen biosynthesis (*.pdf) Click here for file

Additional File 7 **Supplementary **table 6. Protein similarity search of insecticidal toxin of *M. morganii *(*.pdf) Click here for file

Additional File 8 Supplementary table 7. *M. morganii *genes involved in superoxide stress (*.pdf) Click here for file

Additional File 9 Supplementary Table 8. 2 prophages and 12 degenerate prophages (*.xls) Click here for file

Additional File 10 Supplementary Table 9. Specific CDSs in M. morganii compared to other Enterobacteriaceae (n = 14) and/or Proteeae (n = 5). (*.xls) Click here for file

Additional File 11 **Supplementary **Table 10. Comparison of CDSs in M. morganii compared to other Enterobacteriaceae (n = 14) and Proteeae (n = 5). (*.xls) Click here for file
