# Supplementary material for: Whole-genome sequencing and identification of Morganella morganii KT pathogenicity-related genes
Source: BMC Genomics. 2012 Dec 7;13(Suppl 7):S4. doi: 10.1186/1471-2164-13-S7-S4 (PMC3521468; doi:10.1186/1471-2164-13-S7-S4)
Supplement: Additional File 1 — Supplementary Figure 1. The origin of replication was assigned based on the GC deviation of the genome using Ori-Finder (*.pdf) [file 1471-2164-13-S7-S4-S1.pdf]

Supplementary Figure 1. The origin of replication was assigned based on the GC deviation of the genome using Ori-Finder

The information of genome and oriC region	
Genome size	248547 nt
Genome GC content	0.5107
DnaA box distribution	[DnaA box distribution]
OriC length	372 nt
OriC AT content	0.6075
The number of DnaA box	3
The location of oriC region	233393..233764 nt
The location of dnaA gene	-
The extremes of GC disparity	232267 nt (minimum), 197 nt (maximum)
The extremes of AT disparity	9593 nt (minimum), 248442 nt (maximum)
The extremes of RY disparity	238657 nt (minimum), 76 nt (maximum)
The extremes of MK disparity	7325 nt (minimum), 231345 nt (maximum)
Note	Note that the E. coli perfect DnaA box (ttatccaca) was searched for with no more than one mismatch.<br>[Gene list (zcurve1.02)]
Z-curves	[Figure1] [Figure2]
OriC Sequence	The DnaA boxes identified in the below sequence are capitalized and also marked in bold, if any.
	gttaaaccttcattacaaaatgcagtagcgcatcgggggagagaatataactcaactctt<br>gccgtctgaccatgagcggtaaagacgcaggtaattatataaagatctttttatttaaag<br>atctctttattagatcttttattaggatcgcgatcctc**TGTGGATAA**gtgttaaatcccc<br>ataaagatcataaaactgtaaaggatcatttcctgtgaagtaccgttgatcctgttcagt<br>ataagctgggatcaaaacgggtac**TTATTCACA**gggcctgacggagagtgatcttataca<br>gtgaataactacgggttgatcgcaagttgatccccgag**TTATCCACA**gttgattgctgtt<br>tttatcagcaaa
