# Supplementary material for: Whole-genome sequencing and identification of Morganella morganii KT pathogenicity-related genes
Source: BMC Genomics. 2012 Dec 7;13(Suppl 7):S4. doi: 10.1186/1471-2164-13-S7-S4 (PMC3521468; doi:10.1186/1471-2164-13-S7-S4)
Supplement: Additional File 3 — Supplementary table 2. M. morganii genes involved in multidrug efflux genes (*.pdf) [file 1471-2164-13-S7-S4-S3.pdf]

Supplementary table 2. ***M. morganii*** **genes involved in multidrug efflux genes**

**Gene#**	**Gene**	**Description**
MM0215	*mdtL*	Multidrug resistance protein
MM0490-0488	*mdtABC*	Multidrug resistance proteins
MM0499		mitomycin resistance protein McrB
MM0539		glyoxalase/bleomycin resistance protein/dioxygenase
MM0562-0564	*arnBCA*	Polymyxin resistance
MM0594	*mdtK*	multidrug efflux protein
MM0597	*ydhC*	putative MFS family transport protein
MM0651	*emrE*	methyl viologen resistance protein
MM0682	*emrB*	multidrug resistance protein B
MM0683	*emrA*	multidrug resistance protein A
MM1438-1439	*mdlAB*	multidrug transporter membrane\ATP-binding components
MM1447-1449	*acrBAR*	Multidrug efflux and repressor
MM1576	*emrD*	Multidrug resistance protein D
MM1661	*marC*	Membrane efflux protein
MM1814	*mdtG*	Multidrug resistance protein
MM1878		MFS family transporter
MM1891		glyoxalase/bleomycin resistance protein/dioxygenase
MM2166	*mdtH*	Multidrug resistance protein

MM2312-2313	*mdtJI*	Multidrug resistance proteins
MM2407	*mdtB*	Multidrug efflux system subunit MdtB
MM2431		mfs transporter; major facilitator superfamily
MM2434		major facilitator superfamily MFS_1
MM2435		major facilitator superfamily MFS_1
MM2535	*emrA*	multidrug resistance protein A
MM2536	*emrB*	multidrug resistance protein B
MM2968		putative multidrug resistance protein
