# Supplementary material for: Whole-genome sequencing and identification of Morganella morganii KT pathogenicity-related genes
Source: BMC Genomics. 2012 Dec 7;13(Suppl 7):S4. doi: 10.1186/1471-2164-13-S7-S4 (PMC3521468; doi:10.1186/1471-2164-13-S7-S4)
Supplement: Additional File 4 — Supplementary table 3. Flagellum-related genes and chemotaxis genes located in 58.8-kb locus of M. morganii (*.pdf) [file 1471-2164-13-S7-S4-S4.pdf]

## Supplementary table 3. Flagellum-related genes and chemotaxis genes located in 58.8-kb locus of *M. morganii*

| Gene# | Gene | Description |
|---|---|---|
| MM1735 | *fliZ* | flagella biosynthesis protein FliZ |
| MM1736 | *fliA* | flagellar biosynthesis sigma factor |
| MM1737 | *fliC* | flagellin |
| MM1738 | *fliC* | flagellin |
| MM1739 | - | LysR family transcriptional regulator |
| MM1740 | *fliD* | flagellar filament capping protein |
| MM1741 | *fliS* | flagellar protein FliS |
| MM1742 | *fliT* | putative flagellar biosynthesis; export chaperone for FliD |
| MM1743 | *ccrB* | camphor resistance protein CrcB |
| MM1744 | - | CrcB protein |
| MM1745 | - | putative O-antigen polymerase |
| MM1746 | - | lipoprotein |
| MM1747 | - | hypothetical protein |
| MM1748 | - | membrane protein |
| MM1749 | - | hypothetical protein |
| MM1750 | *fliE* | flagellar hook-basal body complex protein flie |
| MM1751 | *fliF* | flagellar MS-ring protein |
| MM1752 | *fliG* | flagellar motor switch protein G |
| MM1753 | *fliH* | flagellar assembly protein H |
| MM1754 | *fliI* | flagellum-specific ATP synthase |
| MM1755 | *fliJ* | flagellar biosynthesis chaperone |
| MM1756 | *fliK* | flagellar hook-length control protein FliK |

| | | |
|---|---|---|
| MM1757 | *fliL* | flagellar basal body-associated protein FliL |
| MM1758 | *fliM* | flagellar motor switch protein FliM |
| MM1759 | *fliN* | flagellar motor switch and energizing protein |
| MM1760 | *fliO* | flagellar biosynthesis protein |
| MM1761 | *fliP* | Flagellar biosynthetic protein fliP |
| MM1762 | *fliQ* | flagellar biosynthesis protein FliQ |
| MM1763 | *fliR* | flagellar biosynthesis protein FliR |
| MM1764 | - | short-chain dehydrogenase/reductase SDR |
| MM1765 | - | putative Transcription factor, LysR family |
| MM1766 | *flgL* | flagellar hook-associated protein 3 |
| MM1767 | *flgK* | flagellar hook-associated protein 1 |
| MM1768 | *flgJ* | flagellar rod assembly protein/muramidase FlgJ |
| MM1769 | *flgI* | putative flagella basal body protein |
| MM1770 | *flgH* | flagellar basal body L-ring protein |
| MM1771 | *flgG* | flagellar basal-body rod protein |
| MM1772 | *flgF* | flagellar basal-body rod protein |
| MM1773 | *flgE* | flagellar hook protein FlgE |
| MM1774 | *flgD* | basal-body rod modification protein |
| MM1775 | *flgC* | flagellar basal body rod protein FlgC |
| MM1776 | *flgB* | flagellar basal body rod protein FlgB |
| MM1777 | *flgA* | basal-body periplasmic P ring assembly protein |
| MM1778 | *flgM* | anti-sigma28 factor |
| MM1779 | *flgN* | flagella synthesis protein |
| MM1780 | *xptA1* | insecticidal toxin complex protein A |

| | | |
|---|---|---|
| MM1781 | *xptA1* | insecticidal toxin complex protein A |
| MM1782 | *xptC1* | insecticidal toxin complex protein B |
| MM1783 | *flhA* | flagellar biosynthesis protein FlhA |
| MM1784 | *flhB* | flagellar biosynthesis protein FlhB |
| MM1785 | - | hypothetical protein |
| MM1786 | *cheZ* | chemotaxis regulator CheZ |
| MM1787 | *cheY* | chemotaxis regulatory protein CheY |
| MM1788 | *cheB* | chemotactic response regulator in two-component regulatory system with CheA |
| MM1789 | *cheR* | chemotaxis methyltransferase CheR |
| MM1790 | *tap* | methyl-accepting chemotaxis protein IV |
| MM1791 | *cheD* | methyl-accepting chemotaxis protein I |
| MM1792 | *cheW* | purine-binding chemotaxis protein; regulation |
| MM1793 | *cheA* | chemotaxis protein CheA |
| MM1794 | *motB* | motility protein B |
| MM1795 | *motA* | proton conductor component of motor, torque generator |
| MM1796 | *flhC* | transcriptional regulator of flagellar class II biosynthesis, anaerobic respiration and the Entner-Doudoroff pathway, tetramer with FlhD |
| MM1797 | *flhD* | transcriptional activator FlhD |

41 flagellum-related genes were in red colors, 8 chemotaxis genes were in green colors, 11 other genes were in blue colors.
