# Supplementary material for: Whole-genome sequencing and identification of Morganella morganii KT pathogenicity-related genes
Source: BMC Genomics. 2012 Dec 7;13(Suppl 7):S4. doi: 10.1186/1471-2164-13-S7-S4 (PMC3521468; doi:10.1186/1471-2164-13-S7-S4)
Supplement: Additional File 5 — Supplementary table 4. Protein similarity search of Type III secretion system (T3SS) of M. morganii (*.pdf) [file 1471-2164-13-S7-S4-S5.pdf]

Supplementary table 4. Protein similarity search of Type III secretion system (T3SS) of *M. morganii*

T3SS genes compare with *P. mirabilis* (15 of 24, BLASTP e-values < 5.0E-05)

T3SS components (MM0224 to MM0243)

MM0225 22%, MM0226 25%, MM0227 54%, MM0231 48%, MM232 43%, MM0233 33%, MM0234 55%, MM0235 37%, MM0236 52%, MM0237 26%, MM0239 24%, MM0240 62%, MM0241 60%, MM0242 40%, MM243 46%

T3SS effector proteins (MM0244 to M0247, chaperons-IpaDBC , operon reverse)

MM0244 (chaperons) 48%

MM0245 (IpaD) 38%

MM0247 (IpaC) 21%

Except for the effector proteins encoding genes, significant best hits were found to the orthologs from *P. mirabilis.* The IpaC and IpaD have low identity (21% and 38%) to those of *P. mirabilis*. %, percent of protein identity in BLAST search.
