# Supplementary material for: Whole-genome sequencing and identification of Morganella morganii KT pathogenicity-related genes
Source: BMC Genomics. 2012 Dec 7;13(Suppl 7):S4. doi: 10.1186/1471-2164-13-S7-S4 (PMC3521468; doi:10.1186/1471-2164-13-S7-S4)
Supplement: Additional File 6 — Supplementary table 5. M. morganii genes involved in lipopolysaccharide or enterobacterial common antigen biosynthesis (*.pdf) [file 1471-2164-13-S7-S4-S6.pdf]

Supplementary table 5. *M. morganii* genes involved in lipopolysaccharide or enterobacterial common antigen biosynthesis

Gene#	Name	Description
MM0018	*wzzE*	lipopolysaccharide biosynthesis protein WzzE
MM0023	*rffC*	putative acyl-CoA N-acyltransferase, lipopolysaccharide biosynthesis protein
MM0024	*rffA*	TDP-4-oxo-6-deoxy-D-glucose transaminase
MM0025	*wzxE*	O-antigen translocase
MM0477	*pagP*	palmitoyl transferase
MM0566	*arnT*	4-amino-4-deoxy-L-arabinose transferase
MM0569	*arnT*	4-amino-4-deoxy-L-arabinose transferase
MM0905	*msbA*	lipid transporter ATP-binding/permease
MM0906	*lpxK*	tetraacyldisaccharide 4'-kinase
MM0909	*kdsB*	CTP:CMP-3-deoxy-D-manno-octulosonate transferase
MM1310	*kdsC*	3-deoxy-D-manno-octulosonate 8-phosphate phosphatase
MM1461	*fepE/wzz*	ferric enterobactin transport protein FepE
MM1593		methyltransferase
MM1813	*htrB/waaM*	lipid A biosynthesis lauroyl acyltransferase
MM1602	*rfaD/waaD*	ADP-L-glycero-D-mannoheptose-6-epimerase
MM1603	*rfaF/waaF*	ADP-heptose
MM1604	*rfaC/waaC*	ADP-heptose:LPS heptosyl transferase I
MM1605		lipopolysaccharide core biosynthesis protein
MM1606	*wabH*	lipopolysaccharide core biosynthesis glycosyl transferase
MM1607	*wabG*	lipopolysaccharide core biosynthesis glycosyl transferase

MM1608	*waaQ/rfaQ*	lipopolysaccharide core biosynthesis glycosyl transferase
MM1609	*waaA*	3-deoxy-D-manno-octulosonic-acid transferase
MM1610	*waaE*	lipopolysaccharide core biosynthesis glycosyl transferase
MM1611	*coaD*	phosphopantetheine adenylyltransferase
MM1613	*rfaL*	O-antigen ligase
MM1614		glycosyltransferase
MM2021	*hldE/rfaE*	bifunctional heptose 7-phosphate kinase/heptose 1-phosphate adenyltransferase
MM2099	*lpxD*	UDP-3-O-[3-hydroxymyristoyl] glucosamine N-acyltransferase
MM2101	*lpxA*	UDP-N-acetylglucosamine acyltransferase
MM2102	*lpxB*	lipid-A-disaccharide synthase
MM2144	*msbB*	lipid A biosynthesis (KDO)2-(lauroyl)-lipid IVA acyltransferase
MM2175	*kdsA*	3-deoxy-D-manno-octulosonic acid 8-P synthetase
MM2266		lipopolysaccharide core biosynthesis protein RfaQ
MM2292	*rfaB*	UDP-D-galactose:(glucosyl)lipopolysaccharide-1,6-D-galactosyltransferase
MM2467	*lpxH*	UDP-2,3-diacylglucosamine hydrolase
MM2978	*pgi*	glucose-6-phosphate isomerase
MM2920	*galU*	glucose-1-phosphate uridylyltransferase
MM3182	*lpxC*	UDP-3-O-[3-hydroxymyristoyl] N-acetylglucosamine deacetylase
MM3398	*galE*	UDP-galactose 4-epimerase

Enterobacterial common antigen

MM0017	*wecA*	undecaprenyl-phosphate alpha-N-acetylglucosaminyltransferase
MM0019	*rffE*	UDP-N-acetyl glucosamine-2-epimerase
MM0020	*wecC*	UDP-N-acetyl-D-mannosamine dehydrogenase
MM0021	*rffG*	dTDP-D-glucose-4,6-dehydratase
MM0022	*rffH*	glucose-1-phosphate thymidylyltransferase
MM0026	*rffT*	4-alpha-L-fucosyltransferase
MM0027	*wzyE*	putative common antigen polymerase
MM0028	*rffM*	UDP-N-acetyl-D-mannosaminuronic acid transferase
