# Supplementary material for: Whole-genome sequencing and identification of Morganella morganii KT pathogenicity-related genes
Source: BMC Genomics. 2012 Dec 7;13(Suppl 7):S4. doi: 10.1186/1471-2164-13-S7-S4 (PMC3521468; doi:10.1186/1471-2164-13-S7-S4)
Supplement: Additional File 7 — Supplementary table 6. Protein similarity search of insecticidal toxin of M. morganii (*.pdf) [file 1471-2164-13-S7-S4-S7.pdf]

Supplementary table 6. Protein similarity search of insecticidal toxin of *M. morganii*

Insecticidal toxin genes:

Gene#	Gene	Species and amino acid identity of three best hits
MM0965	*tccB/*<br>*tccB3*	*Pseudomonas entomophila* L48 35%(coverage:67%)<br>*Burkholderia rhizoxinica* HKI 454 26%<br>Photorhabdus *luminescens ssp. lau.* TTO1 29% (cov. 66%)
MM0966	*tccA3*	*Pseudomonas entomophila* L48 29%<br>*Burkholderia rhizoxinica* HKI 454 29%<br>*Pseudomonas brassicacearum* subsp. *Bra*. NFM421 26%
MM0967	*tcdb2*	*Photorhabdus asymbiotica* 45%<br>*Photorhabdus luminescens* 44%<br>*Photorhabdus luminescens* subsp. laumondii TTO1 44%
MM1780	*xptA1/*<br>*sepA/*<br>*tcdA4*	*Xenorhabdus nematophila* ATCC 19061 35%<br>*Serratia entomophila* 37%<br>*Photorhabdus luminescens* 41% (cov. 67%)
MM1781	*xptA1/*<br>*tcdA4/*<br>*sepA*	*Xenorhabdus nematophila* ATCC 19061 37%<br>*Photorhabdus luminescens* 36%<br>*Serratia entomophila* 40% (cov. 71%)
MM1782	*xptC1/*<br>*sppB/*<br>*sepB*	*Erwinia* sp. Ejp617 51%<br>*Serratia proteamaculans* 50%<br>*Serratia proteamaculans* 49%
MM1901	*tcdA4/*<br>*xptA1/*<br>*tcbA*	*Photorhabdus luminescens* spp. lau. TTO1 41%<br>*Xenorhabdus nematophila* ATCC 1906 56%<br>*Photorhabdus luminescens* 53%
MM2567	*tccB3/*<br>*tccB*	*Pseudomonas entomophila* L48 34%<br>*Salmonella enterica* ssp. *ari.* serovars 32%<br>*Salmonella enterica* ssp. *hou*. 33%
MM2570	*tcaC*	*Burkholderia rhizoxinica* HKI 454 37%

		Bacillus thuringiensis IBL 200 35%<br><br>*Rahnella aquatilis* ATCC 33071 37%
