# Supplementary material for: Whole-genome sequencing and identification of Morganella morganii KT pathogenicity-related genes
Source: BMC Genomics. 2012 Dec 7;13(Suppl 7):S4. doi: 10.1186/1471-2164-13-S7-S4 (PMC3521468; doi:10.1186/1471-2164-13-S7-S4)
Supplement: Additional File 8 — Supplementary table 7. M. morganii genes involved in superoxide stress (*.pdf) [file 1471-2164-13-S7-S4-S8.pdf]

Supplementary table 7. *M. morganii* genes involved in superoxide stress

Gene#	Gene	Description
MM0462	*katA*	Catalase
MM0474	*soxS*	AraC family transcriptional regulator
MM0572	*sodC*	Copper-zinc superoxide dismutase
MM0600	*sodB*	Fe superoxide dismutase
MM1550	*oxyR*	DNA-binding transcriptional regulator OxyR
MM2677	*sodA*	Mn superoxide dismutase
